# Farmer typology to understand differentiated climate change adaptation in Himalaya

**DOI:** 10.1038/s41598-019-56931-9

**Published:** 2019-12-30

**Authors:** Roopam Shukla, Ankit Agarwal, Christoph Gornott, Kamna Sachdeva, P. K. Joshi

**Affiliations:** 1 Department of Natural Resources, TERI University, New Delhi, 110070 India; 2 Potsdam Institute for Climate Impact Research (PIK), Member of the Leibniz Association, Telegrafenberg, Potsdam, 14476 Germany; 3 Department of Hydrology, Indian Institute of Technology, Roorkee, 247667 India; 4 GFZ German Research Centre for Geosciences, Section 4.4: Hydrology, Telegrafenberg, Potsdam, Germany; 5 Spatial Analysis and Informatics Lab (SAIL), School of Environmental Sciences, Jawaharlal Nehru University, New Delhi, 110067 India; 6 Special Center for Disaster Research, Jawaharlal Nehru University, New Delhi, 110067 India

**Keywords:** Mathematics and computing, Physics

## Abstract

Smallholder farmers’ responses to the climate-induced agricultural changes are not uniform but rather diverse, as response adaptation strategies are embedded in the heterogonous agronomic, social, economic, and institutional conditions. There is an urgent need to understand the diversity within the farming households, identify the main drivers and understand its relationship with household adaptation strategies. Typology construction provides an efficient method to understand farmer diversity by delineating groups with common characteristics. In the present study, based in the Uttarakhand state of Indian Western Himalayas, five farmer types were identified on the basis of resource endowment and agriculture orientation characteristics. Factor analysis followed by sequential agglomerative hierarchial and K-means clustering was use to delineate farmer types. Examination of adaptation strategies across the identified farmer types revealed that mostly contrasting and type-specific bundle of strategies are adopted by farmers to ensure livelihood security. Our findings show that strategies that incurred high investment, such as infrastructural development, are limited to high resource-endowed farmers. In contrast, the low resourced farmers reported being progressively disengaging with farming as a livelihood option. Our results suggest that the proponents of effective adaptation policies in the Himalayan region need to be cognizant of the nuances within the farming communities to capture the diverse and multiple adaptation needs and constraints of the farming households.

## Introduction

Agriculture forms the main livelihood source for 70% of the Himalayan region, and it has been a significant contributor to the household food security of the local communities^1,2^. However, the current discourses on agriculture in the Himalaya have recurrently highlighted the on-going agrarian distress in the region manifested by deteriorating land productivity^3^, declining yield in the last decades^4^, exacerbating food insecurity^5^ and deepening poverty^6^. Farmers, in recent times, have been a victim of unprecedented climate-induced social, economic and environmental transitions in the region^7,8^.

The communities inhabiting the fragile mountain regions are known to be disproportionately vulnerable to climate-related changes owing to their high dependence on climate-sensitive agricultural livelihoods^9–11^. In addition, high dependence on monsoons, unavailability of irrigation facilities and small landholdings intensify the sensitivity of Himalayan farmers^12,13^. The climate-related shift in production capacity has led to changes in crop yield, reduced crop diversity and increased pest-invasion in the region^4,14^. Future climatic projections show considerable uncertainty in the magnitude of precipitation and temperature within the region^15,16^. This dynamism induced by ongoing unprecedented environmental shifts has exposed farmers to unusual conditions leading to livelihood and food insecurity^6,9,17^.

Several empirical studies have reported that farmers in the region are adjusting their agricultural practices to adapt to climate-induced agronomic changes^8,18,19^. However, studies in other geographical locations have documented that responses to agronomic risks are socially diverse and spatially distinct, as farmers do not represent a monotypic group and there exists pervasive heterogeneity rooted in differential biophysical, social, economic, cultural and institutional factors^20–22^. These factors together generate differences in resource endowment and production orientation, with a distinctive set of constraints and opportunities thereby creating diversity in their adaptive capacity and choice of adaptive action^23,24^. Therefore, the understanding of farmer diversity is imperative for national, subnational and local policies, seeking to leverage climate change adaptation in the agriculture sector for improving food security and livelihood development of the marginalized Himalayan farmer.

Typology, which refers to the study of types, aims to identify farmers with common characteristics. Farmer typology construction is recognized as an efficient tool to account for farmer diversity and heterogeneity^25,26^. The literature on the farm(er) typology construction uses myriad forms of conceptual approaches and methods, reflective of differing epistemological standpoints^27,28^. The contrast between structural and functional classification is on the basis of the factors to which the diversity is attributed^25^. The structural typology focuses on production factors such as land, livestock or labor; whereas the functional typology is based on the determinants of livelihood orientations, and decisions of farmers^29^. A dichotomy of the two approaches fails to comprehensively account for complex and diverse farming conditions in the Himalayas^30,31^. Moreover, consideration of functional diversity along with structural diversity is essential in the Himalayan landscape as there are shreds of evidence that the livelihood diversification is a norm in these landscapes.

Mostly literature segment farmers/farming households on the basis of a single variable such as landholding, caste, and gender. On the contrary, in farm or farmer typology studies, classification is based on multiple variables, with the selection of the variables based on the objective and locale of the study. The classical work of Schulman and Garrett^32^, and Laurent *et al*.^20^ highlighted the need to address the complex heterogeneity and differentiation that exists within the agrarian communities. Scholars have aimed to characterize farmer diversity with the use of multivariate statistical typology approaches or participatory approaches, in different geographical regions and different agricultural sub-systems such as crops^33,34^, livestock^35^, wetland^36^. Supplementary Information (SI: Table S.1) provides an overview of typology functions used in some of the studies that used a multivariate statistical approach to understand and segment the diversity within farm(er)s.

**Figure 1:**
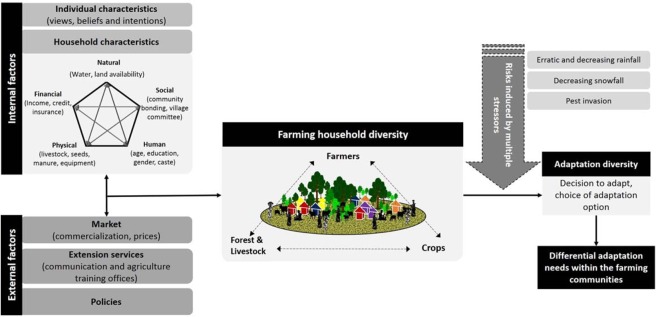
Farming household and adaptation diversity in the Himalayan region. The interplay of internal and external factors generates diversity within farming households. Further household diversity mediates adaptation diversity to agriculture risk leading to differential adaption needs within the farming communities.

Based on the insights gained from literature on farmer typology and Himalayan farming, field experience, and discussion with farmers, we developed a conceptual framework for capturing the drivers of farmer diversity (Fig. 1). The guiding assumption of the framework is that there exist diversity and variability in the Himalayan farming systems in space and time, induced by several internal and external factors. Factors that influence internal diversity to emanate from individual farmer characteristics, such as views, beliefs, and intentions with regards to farming; and household capital characteristics in terms of human, financial, natural, physical, and social capitals^37,38^. Variations in capital and assets base of the household generate differences in structural farming resource endowments, e.g., land, labor, livestock. In addition, access to the market, presence of agriculture extension services like the *Krishi Vigyan Kendra* (KVK, first-line extension agency of Indian Council of Agricultural Research (ICAR)), and farmer welfare and agriculture policies form few of the chief external factors that stimulate diversity. Literature highlights that even low resource endowed households can benefit from proximity to markets and well-integrated extension services which provide access to better farm inputs, e.g., seeds and fertilizers thereby enhancing the agriculture production^39^. The interaction between these internal and external factors generates distinct variations in assets, income, livelihood orientations among farmers.

The present study was undertaken in the Uttarakhand state of Indian western Himalayan region, with an aim to (i) identify predominant farmer types based on the combination of structural and functional attributes, and (ii) examine the adaptive strategies of the identified farmer types to understand the type-specific adaptation choices and emergence of diversified adaptation responses within the farming community. Farmer diversity was defined to be a factor of farmer’s individual and household characteristics, and livelihood orientation. Our results provide the characteristics of identified farmer types along with their adaptation strategies and highlight the need for a cohesive understanding of farmer household diversity for effective adaptation planning that caters to the needs of the different farmers.

## Methods

### Guidelines

All methods were performed in accordance with their relevant guidelines and regulations approved by the institution and funding agency. We adhered to the Code of Ethics of the International Sociological Association (ISA) for the formulation and execution of the questionnaire. The questionnaire was also approved by the institutional committee at *TERI SAS* and pre-tested in the field before the final collection of data. Since the survey was interview-based with humans, before conducting the survey, we informed the participant about the purpose and the utilization of the survey, informed consent was obtained from each of the participants.

### Study area and selection of study villages

A multi-step approach was used to select sites (cluster of villages) in the study area. The selection of study sites was based on the results of a broad scale inherent vulnerability assessment study, executed for the entire state of Uttarakhand^40^. The findings from the hotspot analysis were extended using spatial tools to identify the largest hotspot based on the spatial contiguity of highly vulnerable villages. As a result, Bhikiyasain tehsil in Almora district and Chakrata tehsil in Dehradun district were identified to contain the largest vulnerable patch. Both tehsils lie in the middle altitudinal (1200–1700 m) zone of the Uttarakhand state.

**Figure 2:**
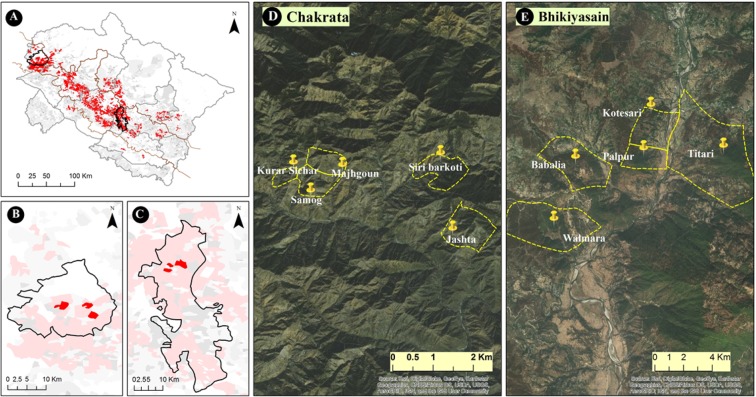
Study villages in Chakrata and Bhikiyasain tehsil. (**A**) Map of inherent vulnerability hotspots for the entire state of Uttarakhand^40^; (**B**,**C**) Distribution of hotspots in Chakrata and Bhikiyasain, respectively; (**D**,**E**) Zoomed in a location of the survey villages. All the maps in Fig. 2 was generated using ArcMap 10.4.1 software. The satellite imagery in figure D and E was generated using default base maps provided by ESRI.

The selection of study villages was made purposively on the basis of three main criteria, total population in the village, share of agriculture population and net cultivated area. Data on these criterions was collected from 2011 census of India. Finally, five villages namely Babalia, Kotesari, Palpur, Titari and Walmara from Bhikiyasain tehsil and five villages namely Kurar Sinchar, Samog, Manjhgaon, Siri barkoti and Jashta from Chakrata tehsil were selected for conducting a household level survey (Fig. 2). Village level appraisal and discussions with key informants (*GramPradhan* (Village Head) and agriculture extension officers) were done to ensure that these villages represent the variation in farming conditions, availability, and accessibility of production resources, and market opportunities which generate diversity within Uttarakhand farming households, so that the established farmer typology can capture maximum heterogeneity in the region. A summary of the land use, crop diversity and socio-economic characteristics of each village is presented in Supplementary Information (SI: Table S.2), along with the crop calendar of various cultivated crops (SI: Table S.3).

### Households (HHs) survey

Primary data collection was done at the household level, which formed the primary sampling unit, over a period of three months from April to June 2017 in the selected 10 villages. A total of 241 HHs were sampled from all the villages. HHs in each of the 10 villages were randomly chosen for questionnaire execution. Survey was divided into two distinct sections, (i) farmer and farm profile that capture the farmer’s personal characteristics, household characteristics, resource endowments, and livelihood orientation, and (ii) documentation of the farmers’ adaptation strategies with regards to crop and cropping pattern changes, livestock management, land-use changes, soil management, infrastructural development, crop insurance, alternate income and food security maintenance. Detailed questions were asked about HH demographic profile, socio-economic status (primary, secondary and tertiary income sources, caste, etc.), agriculture details (land ownership, location of land, crop choice), individual preferences towards farming, and detailed account of HH assets (natural, financial, physical, social, human), food sufficiency and adopted adaptation strategies in response to agriculture risks.

### Variables for typology construction

The selection of variables was done on the basis of the conceptual framework (Fig. 1), context and locale rationale. Farmer diversity was broadly defined to be a function of farmer’s individual characteristics, household characteristics, and production orientation. A total of 29 variables with a combination of numerical (12) and categorical (17) data type was used for farmer typology construction. Table 1 provides a list of the selected variables under each broad dimension.

**Table 1:** List of variables along with their frequencies and mean levels in the case study region (n = 241) and the variables used in factor analysis (FA).

Factor	Variable* [unit]	Included in FA	Data type	Response means/frequencies*
***Farmers personal characteristics***				
	Age (Age) [years]	✓	Continuous	46.49 ± 16.70
	Gender (Gen)	✓	Categorical (nominal)	Female (F): 15.77; Male (M):84.23
	Education (Edu)	✓	Categorical (ordinal)	Illiterate (Ill_Edu): 44.40; Primary (Pri_Edu): 27.39; High School (Hig_Edu): 17.01; Intermediate (Int_Edu): 6.64; Bachelors (Bac_Edu): 3.32; Masters (Mas_Edu): 1.24
	Satisfaction with farming (Sat_Far)	✓	Categorical (ordinal)	Like farming (Lik_Far): 46.04; Dislike farming (Dis_Far): 53.94
	Farming experience [years] (Far_exp)		Continuous	30.93 ± 12.23
	Professionally trained farmer (Tra_Far)	✓	Categorical (binomial)	Yes (Tra_Yes): 18.26; No (Tra_No): 81.74
***Household characteristics***				
Natural asset	Total land holding [ha] (Tot_Land)	✓	Continuous	0.43 ± 0.12
	Percentage of irrigated land (Per_Irr)	✓	Continuous	26.30 ± 13.47
	Percentage of abandoned land (Per_Abn)	✓	Continuous	9.21 ± 2.41
	Location of land (Loc_Land)		Categorical (ordinal)	Upland (Up_lan): 62.87; Lowland (Low_lan): 37.13
Physical asset	Total Livestock Unit (TLU)^b^	✓	Continuous	5.60 ± 1.07
	Ploughing means (Plough)	✓	Categorical (nominal)	Ox (Plo_Ox): 57.26 Power teller (Plo_PT): 8.71 None (Plo_No): 34.02
	Amount of Urea [kg ha^−1^]^c^ (Urea)	✓	Continuous	19.37 ± 11.78
	Amount of DAP [kg ha^−1^]^c^ (DAP)		Continuous	28.30 ± 56.95
	Pesticide usage (Pesti)		Categorical (binomial)	Yes (Pes_Yes): 44.83; No (Pes_No): 55.17
Human asset	Family farm labour (Fam_Lab)	✓	Continuous	3.01 ± 2.00
	Household size (HH_Size)	✓	Continuous	7.65 ± 4.96
	Caste (Cas)	✓	Categorical (ordinal)	General: 40.24; ST: 28.63; OBC: 2.50; SC: 28.63
Financial asset	Household economic status of the (HH_Eco)	✓	Categorical (ordinal)	APL: 39.00; BPL: 61.00
	Months of food sufficiency (Food_Suf)	✓	Categorical (ordinal)	Nil (Nil): 29.46; One (One): 19.10; Three (Three): 32.80; Six (Six): 15.77; Nine (Nine): 2.90
	Usage of hired labour (Hire_Lab)	✓	Categorical (binomial)	Yes (Hir_Yes): 9.54; No (Hire_No): 90.46
	Land tenure (Land_Ten)	✓	Categorical (ordinal)	Self-land owners (Self_Land): 83.82 Self-land as well as rented land (Self_Rent_Land): 9.54 Rented land (Rent_Land): 6.64
	Involvement as daily wage labor (Wage_Lab)	✓	Categorical (binomial)	Yes (Lab_Yes): 34.44; No (Lab_No): 65.56
Social asset	Household members enrolled in village communities (Com_Mem)	✓	Continuous	0.73 ± 0.38
	Perceived social bond (Soc_Bond)	✓	Categorical (ordinal)	Very high (Soc_VH):34.44; High (Soc_H): 23.24; Medium (Soc_M): 17.01; Low (Soc_L): 5.80; Very low (Soc_VL): 19.50
***Production orientation***				
	Major crop sown (Crop)	✓	Categorical (nominal)	Only food crops (Food_Cr): 42.74; More food crops and less cash crops (MFLC): 13.69; More cash crops and less food crops (MCLF): 28.63; Only cash crops (Cash_Cr): 9.54; None (No_Cr): 5.39
	Percentage of income from crop sales (Inc_Crop)		Continuous	38.05 ± 28.28
	Access to agriculture loan/credit (Credit)	✓	Categorical (binomial)	Yes (Cre_Yes): 30.71; No (Cre_No): 69.29
	Availability and access to KVK (KVK)		Categorical (binomial)	Yes (KVK_Yes): 39.09; No (KVK_No): 60.91
	Availability and access to market (Market)	✓	Categorical (binomial)	Yes (Mar_Yes): 46.89; No (Mar_No): 53.11

^*^Characters in the brackets refer to the coded acronym for each category of a categorical variable.

^a^Personal characteristic of the household head was collected as most of the farming decisions are taken by the household head.

^b^Conversion factor: Buffalo-1.5, Bullock-1.2, Cow-1.0, mule/horse-1.0, Cow-calf-0.5, Buffalo calf-0.75, goat-0.2, sheep-0.2 (Singh and Naik, 1987)

^c^Estimate based on the amount brought by a household in one season which is used for all crops.

### Statistical analysis

Typology construction is a sequential and iterative process that involves the following steps: (i) exploratory analysis (outlier analysis, variable transformation, and correlation analysis); (ii) factor analysis and (iii) cluster analysis; (iv) assessing the reliability of clustering results^27,41^. Earlier studies have either divided the continuous data into a category or converted categories to discrete numbers, when dealing with a dataset that originally has mixed variables, to meet the data requirement restrictions imposed by statistical methods^42^. Such procedures result in loss of information and diminish the variability in the data thereby affect the quality of the results^43^. Therefore, our analysis retains the variables in the original type and makes use of mixed data analysis methods dedicated to exploring both categorical and continuous datasets simultaneously. All the statistical analysis and graphical output were obtained using R software (version 3.3.2) using several packages.

#### Exploratory analysis

Exploratory data analysis was done through plotting histograms, boxplot and frequency analysis for detection of an outlier, identification of variable distribution and estimation of “strong” correlations. As multivariate analysis methods are sensitive to the presence of outliers, we initially screened continuous variables for presence outliers using box-plots that were winsorized (top 95^th^ percentile). Square-root and the log-normal transformation were done for few highly skewed continuous variables such as total land, amount of urea, TLU and percentage of abandoned land. Finally, the correlation matrix was computed in R using the *hetcor* function of ‘polycor’ package owing to the mixed type of variables. The *hetcor* function calculates Pearson product-moment between numeric variables, polyserial correlations between numeric and ordinal variables and polychoric correlations between ordinal variables. Based on the output of the *hector* functions variables with greater than 0.9 statistically significant correlation values were removed.

#### Factor analysis on mixed data (FAMD)

Factor analysis is a data reduction technique that allows for reducing a large number of variables into uncorrelated factors that have greater explanatory power. We used the *FAMD* function of the ‘FactoMineR’ package^42^, which is a mix of principal component analysis (PCA) and multiple correspondence analysis (MCA) to simultaneously handle both continuous and categorical data. More details on the assumptions and mathematical derivatives are documented in Husson *et al*.^44^. Factor scores of the factors with an eigenvalue greater than one (as per the Kaiser criterion) were used as input data for cluster analysis to generate typology.

#### Cluster analysis

With the objective of maximizing both intra-cluster homogeneity and inter-cluster heterogeneity, a two-step clustering approach was used to identify the farmer types. Hierarchical Clustering on Principal Components (HCPC) function of ‘FactoMineR’ package was used to perform hierarchical, agglomerative clustering and K-means clustering on the factor analysis output. A hierarchical tree is generated using the hierarchical, agglomerative clustering algorithm using Ward’s method and Euclidean distance matrix. K-means preprocessing consolidation is done after the hierarchical clustering. The minimum and the maximum number of required clusters need to be provided as an input, but the suggested cluster partition is the one with the higher relative loss of inertia (i (clusters n + 1)/i (cluster n)). Along with the subjective inspection of the dendrogram, supported by statistics of inertia gain, we also determined the number of clusters retain using the ‘NbClust’ package for hierarchical clustering (‘ward.D’ method). ‘NbClust’ provided a summary of 23 indices, the number suggested by maximum indices was used to determine the optimal number of clusters. After the identification of farmer types, adaption strategies were listed and compared for each farmer type to understand the diversity in adaptation practices in the region.

## Results and discussion

### Underlying factors of farm typolongy

Table 1 provides the mean and standard deviation of continuous variables and frequencies of various categories of categorical selected variables. Distribution of total land, percent abandoned the land, TLU, and amount of urea and DAP was highly skewed, indicative of the unequal distribution of essential production inputs in the sampled region. Therefore, these variables were transformed using log-normal transformation to attain a near-normal distribution. The results of the correlation analysis revealed that there was strong correlation between percent irrigated land and location of the land (0.99, p-value = *0*.*05*), professional training of farmer with availability and access to KVK (0.96, *0*.*05*), farming experience of farmer and her/his age (0.93, p-value = *0*.*05*), pesticide used and major crops sown (0.92, p-value = *0*.*05*), amount of urea and amount of DAP used (0.92, p-value = *0*.*05*), and percentage income from crop sales and major crop sown (0.91, p-value = *0*.*05*). Thus, farming experience, location of land, access, and availability of KVK, and percent income from crop sales were not included in factor analysis. Table 1 lists the variables that were included in subsequent factor analysis.

**Figure 3:**
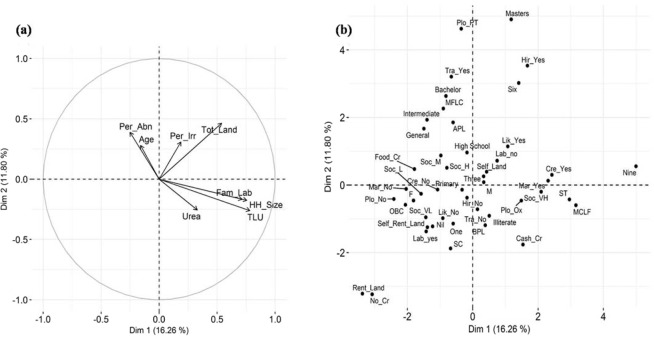
FAMD (PCA and MCA) output. (**a**) Correlation circle represents the loading of continuous variables on the 1 and 2 dimensions; (**b**) Categorical variables factor map projects the class of variables in the plane of 1 and 2 dimensions. Details of the acronym provided in Table 1.

Mixed data factor analysis resulted in the extraction of 13 dimensions, with Eigenvalue greater than one, which explained a total of 67.96% variance of the entire dataset. The first and the second dimension explained 16.26% and 11.80% variability of the data, respectively. The details of Eigenvalue and percentage variance explained by 13 dimensions using FAMD output are provided in the supplementary information (SI: Table S.4). Figure 3 shows the loading of different variables and categories on the first and the second dimension. First dimension was closely related to TLU *(TLU* = *0*.*61*), household size *(HH_Size* = *0*.*57*), family labour *(Fam_Lab* = *0*.*51*), and is discriminated on the basis of categories of market availability *(Market* = *0*.*72*) and crop type grown *(crop* = *0*.*72*) (Fig. 3). Thus, the first dimension seemed to explain human capital and livestock capital and the production orientation of the farmer explained by the choice of crop grown and the availability of the market. The second dimension was explained by percentage of abandoned land *(Per_Abn* = *0*.*42*) and percentage irrigated land *(Per_Irr* = *0*.*34*) and is discriminated based on the categories of trained farmer *(Tar_Far* = *0*.*49*), economic status of the household *(HH_Eco* = *0*.*47*), caste *(Caste* = *0*.*46*), and ploughing means *(Plough* = *0*.*43*). The loading of each variable on the extracted dimension is provided in the supplementary information (SI: Table S.5). It is important to note here that since the *FAMD* function uses a mix of PCA and MCA methods, the variable contribution values tend to be low. However this does not necessarily reflect unsatisfactory results.

**Figure 4:**
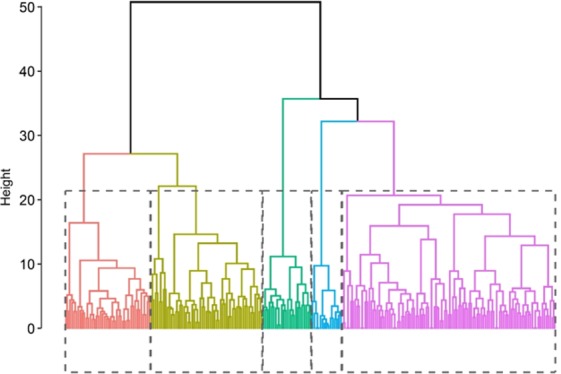
Dendrogram of individuals from the resulting from agglomerative hierarchical clustering of farmer households.

The factor score generated for the 13 dimensions for the 241 farming households was used as input data for cluster analysis. The choice of the optimum number of the cluster was made relative to the inertia gain values and the majority number of clusters suggested by the indices result of the ‘NbClust’ package. Inertia gain plot in the supplementary information (Fig. SI: 1(a)) shows a distinct break after 5 clusters. In addition, out of the 23 indices, 8 suggested to partition the data into 5 clusters, (Figure SI: 1 (b)) therefore, the farming households were grouped into 5 clusters. The dendrogram of the individuals generated from agglomerative hierarchical clustering is shown in (Fig. 4).

### Characterization of each farmer type

**Figure 5:**
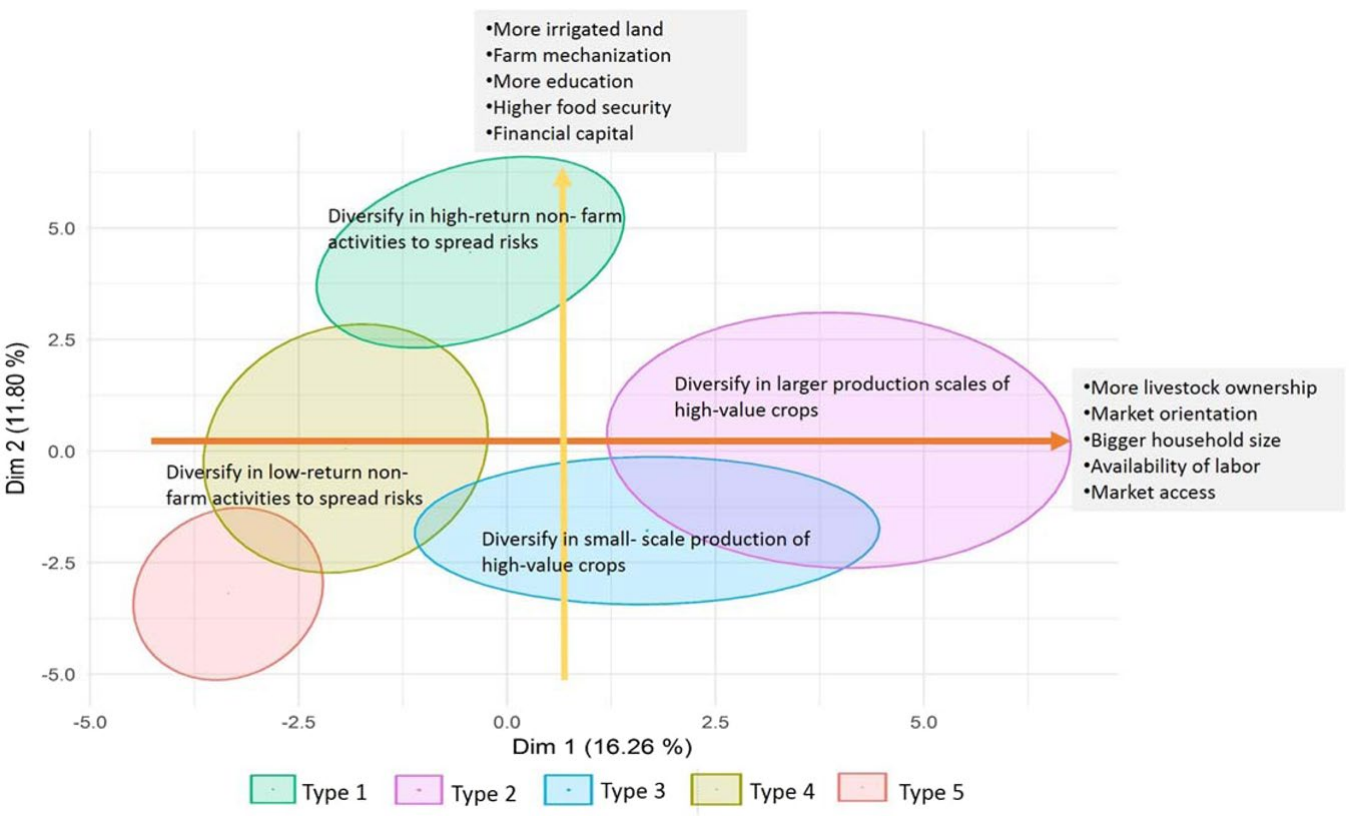
Distribution of the surveyed household in five farmer household types on the 1 and 2- dimensional plane.

We characterized the farmer types (clusters) based on the farmer’s resource and capital endowment along with individual and production orientation, details of which are provided in the supplementary information (SI: Table S.6) and (SI: Table S.7). Table S.6 lists mean and standard deviation of continuous variables for each of the farmer type clusters and Table S.7 provides estimates of specificities and homogeneities for all farmer type groups. Further, Fig. 5 shows the distribution of five different farmer types and broadly summarizes the main factors that differentiate identified farmer adaptation strategies. The specific characteristics of each farmer type are discussed in the sub-sections below.

#### Type 1: High resource-endowed: intensified food crop farmers (n = 29, 12%)

Type 1 cluster represents the group of farmers having greater irrigated cultivated land, larger land holding, mainly growing food crops by making excessive use of power teller and hired labor. This farmer group has strong financial capital with most of the income being generated from salaried jobs and absolute non-involvement in labor work. All the farmers in this cluster belong to the upper caste (general category) with income status Above Poverty Line (APL). The mean age of head of the HH in this group is approximately 58 years with relatively small household size, less family labor, and livestock unit. Although most of the farmers in this cluster reported to like farming, yet the rate of land abandonment is the highest. These farming households have intensified the production of food crops through the use of external knowledge gained through training and usage of improved seeds from the KVKs. However, the availability of family farm labor is low as most of the members of the household have migrated. Therefore labor availability becomes the main constraint for this farmer type that is mitigated through hiring non-family labor and these are abandoning agriculture lands located far from residential household sites.

#### Type 2: High resource-endowed: market-oriented cash crop farmers (n = 43, 18%)

Type 2 cluster farmers are landowners with the largest landholding and highest TLU compared to the rest of the farmer types, characterized by a large household member, sizable family labor, and large irrigated fields mostly cultivated with cash crops (SI: Table S.6). Income is generated mostly through selling cash crops due to the easy accessibility and availability of the market. Ploughing of fields is mostly done using Ox. Food grain sufficiency (months/year) is also comparatively better for the Type 2 group members with about 50% of the household with at least six months of food grain sufficiency. Further, most of these farmers have access to credits. Type 2 farmers utilize the availed credit for predominantly buying HYV seeds of cash crops mostly, vegetables, thereby shifting the focus from traditional cereal crops to high-value cash crops.

#### Type 3: Medium resource-endowed: market-oriented cash crop farmers (n = 53, 22%)

Type 3 farmer clusters are characterized by young to mid-aged household heads who only grow cash crops with extensive usage of fertilizers along with extensive market integration (SI: Table S.6). Agriculture landholding is low with less irrigated fields. However, due to the larger family size, the enhanced availability of family farm labor increases the economic viability of farms for cultivating cash crops. The group members are from both ST (Scheduled Tribes) and SC (Scheduled Castes). With regard to individual preferences, about 74% of the farmers in this cluster don’t like farming. The months of food grain sufficiency is low (<3 months/year) for about 80% of the farmers. Regarding the income status of the household, 97% of households in this group fall below the poverty line. Thus Type 3 farmers are constrained by financial capacity which affects timely access of agriculture inputs, adequate access to equipment for transport, and adoption of any new technology thereby productivity and profitability is not well maintained.

#### Type 4: Low resource-endowed: subsistence food crops framers (n = 100, 41%)

The maximum number (41%) of the surveyed households was classified under the Type 4 category. Type 4 farmer type is constrained with small landholdings, limited availability of farm labor and smaller livestock unit and household size. Most importantly, about 70% of the farmers in this type do not own any means of ploughing i.e., these households neither use any mechanized instrument such as power teller nor have an ox in their livestock composition. Although farm household uses external fertilizers, the amount remains considerably low as compared to other high and medium endowed farmer types. Market access and availability and credit facilities are also restricted. About 60% of the income of these farmer types is generated from low paying manual labor work, therefore, these farmers do not have the financial capacity to overcome the labor constraint by employing hired labor. Intriguingly, all the women-headed households present in the entire dataset were grouped under this cluster. This is indicative of the poor resource conditions that the women-headed households are currently living in and the greater resource constraints these households face.

#### Type 5: Low resource-endowed: disengaging farm labours (n = 16, 7%)

7% of the surveyed households were classified as Type 5 farmers, low resource-endowed: disengaging farmers. This smallest group of farmers represents the most disadvantaged group of farmers concerning all forms of household capital such as land, TLU, farm labor, usage of fertilizers, and availability of credits. The households classified in this cluster are mostly landless farmers who work as farm laborers, mostly belonging to SC (93%) and falling in Below Poverty Line (BPL) category. Regarding production orientation, all farmers in this cluster have disengaged from farming, and do not prefer sharecropping or farm labor as they don’t see farming as a desirable option anymore. Most of the farmers work as daily wage laborers in unskilled sectors.

### Diversity in adaptation strategies

**Figure 6:**
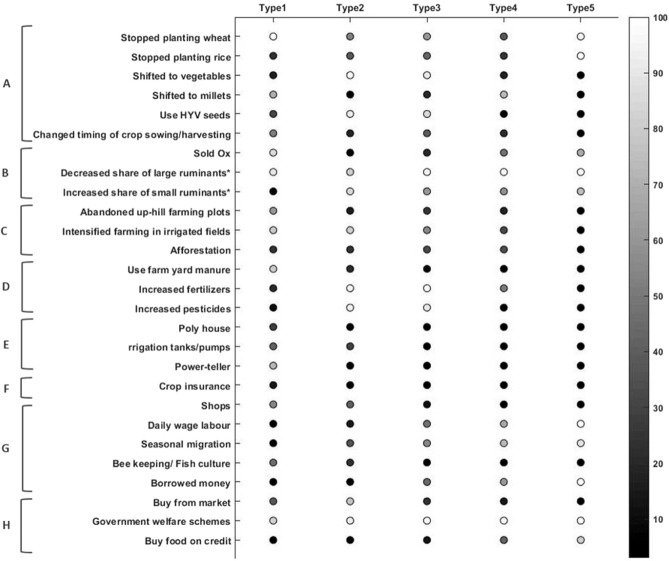
Percentage distribution of different adaptation strategies across five farmer types. (**A**): crop and cropping pattern changes; (**B**): Livestock composition changes; (**C**): Land use changes; (**D**): Soil management; (**E**): Infrastructure development; (**F**): Crop insurance; (**G**): Alternative income sources; H: Food Security maintenance. (*Large ruminants include cattle and buffaloes, whereas the small ruminants include goats and poultry).

This section reports the adaptive strategies opted by each farmer type in response to the environmental, specifically climate change-induced stressors. The results show that farmers are not merely passive victims to agriculture challenges, rather respond actively through the adoption of a bundle of so-called adaptation strategies like crop and cropping pattern changes, livestock changes, land-use changes, soil management, infrastructural development, crop insurance, taking up of alternative livelihood options and food security maintenance (Fig. 6). Five different categories of adaption strategies, specific for each farmer type, were defined following the approach proposed by Dorward’s^45^ tripartite schema of livelihood pathway ‘hanging-in’, ‘stepping-up’, and ‘stepping-out’; further extended by Scoones *et al*.^46^ into quadripartite classification.

#### Type 1: ‘Stepping out’ – ‘Stepping-up’ strategy

Type 1 i.e., the high resource-endowed farmers with subsistence orientation reported a continuum of adaptation strategies that could be broadly classified as ‘stepping-out’ – ‘stepping-up’. Overall, Type 1 farmers reported the maximum adoption of soil management, infrastructure development, and crop insurance mechanisms. Shops (55.17%) and, bee-keeping/fish culture (44.82%) were alternative income sources that helped the farmers compensate for climate-induced agriculture losses. As the main orientation of this farmer group is subsistence-based, in accordance, the results indicate that farmer Type 1 has mostly shifted to growing millets (68.96%), as millets were reported to be stress-tolerant and aided in securing household food security. Moreover, the farmers have adjusted their timing of crop sowing and harvesting in response to climatic stressors. Overall there is a decline in livestock size, and farmers in this category have sold away ox (86.20%) in turn adopting mechanized power-teller for ploughing agricultural fields. As identified in the previous section, this farmer type represents farmers who have well-paying salaried jobs, therefore, financial capital is not a constraint.

#### Type 2: ‘Stepping-up’ strategy

Farming forms the core activity that supports their livelihood and income of Type 2 farmers. These farmers have sufficient land and labor resources that are utilized extensively to meet the needs of a market-oriented outlook on farming activities. All the farmers have altered their crop choices and now predominantly grow vegetables (100%) with intensified usage of HYV seeds (90.70%) and chemical fertilizers (95.34%). None of the households in this cluster have sold ox, as Type 2 farmers identify the critical benefits of a hand-drawn ox ploughing for enhancing the productivity of soil through maintaining soil quality. Similar to Type 1 farmers, financial capital is not a constraint, and hence a portion of these farmers invest in irrigation infrastructural development as well (30.23%).

#### Type 3: ‘Hanging-in’ strategy

Type3 farmers show considerable variation in crop choices with shifting focus from food to cash crops (92.45%), which require intensive fertilizers (98.11%) and pesticide inputs (92.41%). The higher number of farmers in this group seeks seasonal migration (54.71%), work as daily wage labor (47.16%) and borrowed money (43.39%) to meet the financial needs of the household. Further, household food security is mainly met through PDS sources (100%) with a few households also relying on the market (22.64%) and informal credit (13.20%). Highly dynamic land-use changes are also observed in this category of the farmer as 24.52% of the farmers have abandoned up-hill farming plots, and a greater share of farmers prefer to intensively grow vegetables on irrigated fields (54.72%).

#### Type 4: ‘Hanging-in’ – ‘Dropping-out’ strategy

The low resource endowed subsistence orientation farmers form the Type 4 farmer also show a distinct pattern of crop choice changes. A greater percentage of farmers prefer to grow millets (73%) as compared to all other farmer types. In addition, about 37% and 25% of the farmers have stopped cultivating wheat and rice, respectively. Widespread changes were also reported in the livestock composition with decreasing share of large ruminants and increasing share of small ruminants, in conjugation with the selling of ox by 47% of the households. The use of capital-intensive soil and infrastructure development strategies was also insufficient in farmers of this type and household financial needs were mostly met through working as daily wage labor (67%), seasonal migration (73%) and borrowing money (62%).

#### Type 5: ‘Dropping out’ strategy

Type5 farmers represent destitute farmers, constrained by unavailability of farming resources and other endowments. Almost all the farmers in this type have exited farming (SI: Table S.7). 93.75% and 100% of the farmers have stopped cultivating wheat and rice, respectively. About 70% of the farmers have sold ox and presently do not own any tool for ploughing fields. Based on the response of the farmers in this type, farmers have ceased the farming practice and thereby are entirely dependent on government welfare schemes, such as Public Distribution System (PDS) of the government of India to meet household food security.

### Policy implication for addressing differential adaptation needs

While comparing similar research from different regions, it is evident that there exists distinct heterogeneity among farmers across the mountain regions^10^. The heterogeneity is institutionalized with respect to differential farmer endowments, farming preferences and orientation and these farm differentials lead to diversified adaptation responses^22,47^. The results show that farmer Types with strong market linkages, greater assets, larger family and livestock size are more capable of ‘stepping-up’ and adapt to changes. It can be interpreted that social and economic inequalities are being further reinforced resulting in differential upward and downward mobility of these farmer types. For instance, the Type 5 farmer showcases a downward spiral leading in discernible adaptation deficit and recurring patterns of hardship. This is one reason why agriculture policies have not been able to mainstream climate change adaptation is the fact that they do not make any rigorous interventions concerning specific agriculture needs. Even though there are existing safeguards in policy like the various types of insurance policies, access to credit, irrigation policies and food aid, the outcomes have been below the desirable levels in the Uttarakhand state^48,49^. Despite many farmers gaining support from these developments, it is likely that others may be affected adversely. In many cases, it is observed that these ‘one-size-fits-all’ approaches have only led to the reinforcement of inequalities among different farmer groups owing to existing power dynamics and socio-cultural dimensions^50^. A single type of risk assistance will not result in equitable, efficient and sustainable outcomes in the long-term.

## Conclusion

The purpose of the paper was to develop the typology of farming household and their relationship with adaptation strategies to climate change. This is based on both the personal and household characteristics as well as the production orientation of the household in the Uttarakhand state of western Indian Himalaya. In order to capture the heterogeneity, a systematic statistical method for typology construction was used which segmented the 241-surveyed household into five farmer types. Based on the results of the study, the land size was not the primary driver of the farming household diversity instead farming labor availability (determined by household size), livestock unit and market availability were identified as the key driver of the household diversity. It is challenging to fully capture the diversity encountered in the farming system and the limitations posed by typology methods. Further the identified farmer types are time-specific, however, given the dynamical nature of agriculture communities and their decision making one type of farmer could transform into another with time. Given these acknowledged limitations, the results of our study comprehensively covered the entire spectrum of the farmers along the continuum of resource endowment (Low to high) and production orientations (subsistence to market-oriented) in the region. The study also reports the importance of farmer types in generating diversity in adaptation strategies and needs in the region. Therefore, the findings of the study provides an entry point to analyze a mix of policy options which aim at curtailing type-specific constraints which act as barriers for each farmer type from adopting welfare-oriented adaptation options thereby negating the concept of ‘one-size-fits-all’ solution for Himalayan farmers rather promoting the notion of ‘adaptation bundle’ of different options.

## Supplementary information

Supplementary information.

## References

[1] Tiwari PC, Joshi B (2015). Local and regional institutions and environmental governance in Hindu Kush Himalaya. Environ. Sci. Policy.

[2] Hussain Abid, Rasul Golam, Mahapatra Bidhubhusan, Tuladhar Sabarnee (2016). Household food security in the face of climate change in the Hindu-Kush Himalayan region. Food Security.

[3] Ojha HR (2017). Agricultural land underutilisation in the hills of Nepal: Investigating socio-environmental pathways of change. J. Rural Stud..

[4] Negi, G. C. S. *et al*. Impact of climate change on the western Himalayan mountain ecosystems: An overview. **53**, 345–356 (2012).

[5] Gautam Y, Andersen P (2016). Rural livelihood diversification and household well-being: Insights from Humla, Nepal. J. Rural Stud..

[6] Gentle P, Maraseni TN (2012). Climate change, poverty and livelihoods: Adaptation practices by rural mountain communities in Nepal. Environ. Sci. Policy.

[7] Barua Anamika, Katyaini Suparana, Mili Bhupen, Gooch Pernille (2013). Climate change and poverty: building resilience of rural mountain communities in South Sikkim, Eastern Himalaya, India. Regional Environmental Change.

[8] Macchi Mirjam, Gurung Amanda Manandhar, Hoermann Brigitte (2014). Community perceptions and responses to climate variability and change in the Himalayas. Climate and Development.

[9] Xu J, Grumbine RE (2014). Building ecosystem resilience for climate change adaptation in the Asian highlands. Wiley Interdiscip. Rev. Clim. Chang..

[10] Gerlitz J, Banerjee S, Brooks N, Macchi M (2015). Handbook of Climate Change Adaptation..

[11] Satyal Poshendra, Shrestha Krishna, Ojha Hemant, Vira Bhaskar, Adhikari Jagannath (2017). A new Himalayan crisis? Exploring transformative resilience pathways. Environmental Development.

[12] Kuniyal JC (2003). Regional imbalances and sustainable crop farming in the Uttaranchal Himalaya, India. Ecol. Econ..

[13] Rasul GF (2014). water, and energy security in South Asia: A nexus perspective from the Hindu Kush Himalayan region☆. Environ. Sci. Policy.

[14] Kaul Vaibhav, Thornton Thomas F. (2013). Resilience and adaptation to extremes in a changing Himalayan environment. Regional Environmental Change.

[15] Choudhary A, Dimri AP (2017). Assessment of CORDEX-South Asia experiments for monsoonal precipitation over Himalayan region for future climate. Clim. Dyn..

[16] Sanjay Jayanarayanan, Krishnan Raghavan, Shrestha Arun Bhakta, Rajbhandari Rupak, Ren Guo-Yu (2017). Downscaled climate change projections for the Hindu Kush Himalayan region using CORDEX South Asia regional climate models. Advances in Climate Change Research.

[17] Panthi Jeeban, Aryal Suman, Dahal Piyush, Bhandari Parashuram, Krakauer Nir Y., Pandey Vishnu Prasad (2015). Livelihood vulnerability approach to assessing climate change impacts on mixed agro-livestock smallholders around the Gandaki River Basin in Nepal. Regional Environmental Change.

[18] Jones HP, Hole DG, Zavaleta ES (2012). Harnessing nature to help people adapt to climate change. Nat. Clim. Chang..

[19] Khanal U, Wilson C, Hoang V-N, Lee B (2018). Farmers’ Adaptation to Climate Change, Its Determinants and Impacts on Rice Yield in Nepal. Ecol. Econ..

[20] Laurent C, van Rooyen CJ, Madikizela P, Bonnal P, Carstens J (1999). Household Typology for Relating Social Diversity and Technical Change. Agrekon.

[21] VanDerWal J, Shoo LP, Johnson CN, Williams SE (2009). Abundance and the environmental niche: environmental suitability estimated from niche models predicts the upper limit of local abundance. Am. Nat..

[22] Tittonell P (2010). The diversity of rural livelihoods and their influence on soil fertility in agricultural systems of East Africa - A typology of smallholder farms. Agric. Syst..

[23] Wood SA, Jina AS, Jain M, Kristjanson P, DeFries RS (2014). Smallholder farmer cropping decisions related to climate variability across multiple regions. Glob. Environ. Chang..

[24] Lyle G (2015). Understanding the nested, multi-scale, spatial and hierarchical nature of future climate change adaptation decision making in agricultural regions: A narrative literature review. J. Rural Stud..

[25] Tittonell P, Leffelaar PA, Vanlauwe B, van Wijk MT, Giller KE (2006). Exploring diversity of crop and soil management within smallholder African farms: A dynamic model for simulation of N balances and use efficiencies at field scale. Agric. Syst..

[26] Karantinini K, Zylbersztajn D (2007). The global farmer: typology, institutions and organisation. J. Chain Netw. Sci..

[27] Alvarez, S., Paas, W., Descheemaeker, K., Tittonell, P. & Groot, J. Typology construction, a way of dealing with farm diversity General guidelines for Humidtropics. *Rep*. *CGIAR Res*. *Progr*. *Integr*. *Syst*. *Humid Trop*. **Plant Scie**, 1–37 (2014).

[28] Kuivanen KS (2016). Characterising the diversity of smallholder farming systems and their constraints and opportunities for innovation: A case study from the Northern Region, Ghana. NJAS - Wageningen J. Life Sci..

[29] Cortez-Arriola J (2015). Leverages for on-farm innovation from farm typologies? An illustration for family-based dairy farms in north-west Michoac??n, Mexico. Agric. Syst..

[30] Shukla R (2019). Climate change perception: an analysis of climate change and risk perceptions among farmer types of Indian Western Himalayas. Climatic Change..

[31] Pandey, R. & Jha, S.K. Climate Vulnerability Index—Measure of Climate Change Vulnerability to Communities: A Case of Rural Lower Himalaya, India. Mitigation and Adaption Strategies, Global Change, 17, 487-506 (2012).

[32] Schulman M, Garrett P (1990). Cluster analysis and typology construction: The case of small-scale tobacco farmers. Sociol. Spectr..

[33] Tittonell P, Vanlauwe B, de Ridder N, Giller KE (2007). Heterogeneity of crop productivity and resource use efficiency within smallholder Kenyan farms: Soil fertility gradients or management intensity gradients?. Agric. Syst..

[34] Iraizoz B, Gorton M, Davidova S (2007). Segmenting farms for analysing agricultural trajectories: A case study of the Navarra region in Spain. Agric. Syst..

[35] Martin-Collado D, Soini K, Mäki-Tanila A, Toro MA, Díaz C (2014). Defining farmer typology to analyze the current state and development prospects of livestock breeds: The Avile??a-Negra Ib??rica beef cattle breed as a case study. Livest. Sci..

[36] Sakané N, Becker M, Langensiepen M, Van Wijk MT (2013). Typology of smallholder production systems in small east-African wetlands. Wetlands.

[37] Aase, T. H., Chapagain, P. S. & Tiwari, P. C. Farming Innovation as an Expression of Adaptive Capacity to Change in Himalayan Farming. **33**, 4–10.

[38] Agarwal, B. *Twelfth Plan Working Group on Disadvantaged Farmers*, *Including Women* (2011).

[39] Skjeflo S (2013). Measuring household vulnerability to climate change—Why markets matter. Glob. Environ. Chang..

[40] Shukla R, Sachdeva K, Joshi PK (2016). Inherent vulnerability of agricultural communities in Himalaya: A village-level hotspot analysis in the Uttarakhand state of India. Appl. Geogr..

[41] Weltin M (2017). Analysing behavioural differences of farm households: An example of income diversification strategies based on European farm survey data. Land use policy.

[42] Lê S, Josse J, Husson F (2008). **FactoMineR**: An *R* Package for Multivariate Analysis. J. Stat. Softw..

[43] Quinn KM (2004). Bayesian Factor Analysis for Mixed Ordinal and Continuous Responses. Polit. Anal..

[44] Husson, F., Lê, S. & Pagès, J. *Exploratory Multivariate Analysis by Example Using R Introduction to Data Technologies* (2011).

[45] Dorward A (2009). Integrating contested aspirations, processes and policy: Development as hanging in, stepping up and stepping out. Dev. Policy Rev..

[46] Scoones I (2012). Livelihoods after land reform in zimbabwe: Understanding processes of rural differentiation. J. Agrar. Chang..

[47] Douxchamps S (2016). Linking agricultural adaptation strategies, food security and vulnerability: evidence from West Africa. Reg. Environ. Chang..

[48] Kelkar U, Narula KK, Sharma VP, Chandna U (2008). Vulnerability and adaptation to climate variability and water stress in Uttarakhand State, India. Glob. Environ. Chang..

[49] Pandey R (2015). Socio-ecological Vulnerability of Smallholders due to Climate Change in Mountains: Agroforestry as an Adaptation Measure. Chang. Adapt. Socio-Ecological Syst..

[50] Sugden F (2014). Agrarian stress and climate change in the Eastern Gangetic Plains: Gendered vulnerability in a stratified social formation. Glob. Environ. Chang..

