# Supplementary material for: Farmer typology to understand differentiated climate change adaptation in Himalaya
Source: Sci Rep. 2019 Dec 30;9:20375. doi: 10.1038/s41598-019-56931-9 (PMC6937272; doi:10.1038/s41598-019-56931-9)
Supplement: Supplementary file 1 — Supplementary information. [file 41598_2019_56931_MOESM1_ESM.docx]

**Farmer typology to understand differentiated climate change adaptation in Himalaya**

**Roopam Shukla^1, 2*^, Ankit Agarwal^2,3,4^, Christoph Gornott^2^, Kamna Sachdeva^1^ and P K Joshi^5,6^**

^1^Department of Natural Resources, TERI University, New Delhi – 110070, India

^2^Potsdam Institute for Climate Impact Research (PIK), Member of the Leibniz Association, Telegrafenberg, Potsdam – 14476, Germany

^3^Department of Hydrology, Indian Institute of Technology, Roorkee-247667, India

^4^GFZ German Research Centre for Geosciences, Section 4.4: Hydrology, Telegrafenberg, Potsdam, Germany

^5^Spatial Analysis and Informatics Lab (SAIL), School of Environmental Sciences, Jawaharlal Nehru University, New Delhi – 110067, India

^6^Special Center for Disaster Research, Jawaharlal Nehru University, New Delhi – 110067, India

*Corresponding author: shukla@pik-potsdam.de

**Supplementary Information**


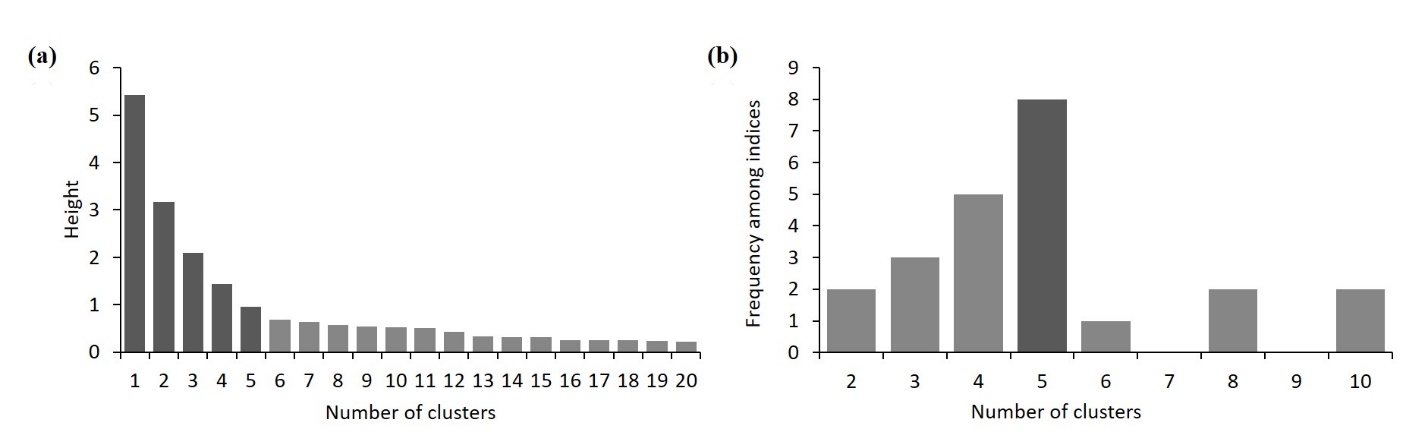


**Figure S.1: Optimum number of cluster for delineating farmer types. (a) Inertia gain plot (b) optimum number of cluster suggested by majority indices.**

**Table S.1: Review of list of factors that have been used in existing literature to segment and characterize heterogeneity in farming systems *(FT = farm(er)s type)***

| **S.No** | **Aim of the study** | **Typology function** | **Reference** |
| --- | --- | --- | --- |
| 1 | Classification of farm household on the basis of their potential access to resources for soil management | FT = f (wealth class, production orientation, farming constraints, household position in farm cycle, family structure, income source) | 1 |
| 2 | Segmentation of farms for analyzing agricultural trajectories | FT = f (farm size, rented land, hired labor, farm specialization, intensification, debt and financial stress, geographic location) | 2 |
| 3 | To symbolize farm households based different socio-economic factors that influence adoption of technological interventions | FT = f (farm size, education, risk perception and risk attitude, income, labor availability, land ownership, personal attributes of household head, technological attributes) | 3 |
| 4 | To construct the typology of households in small wetlands using combination of production system attributes land resources and production objectives | FT = f (production resources and production orientation) | 4 |
| 5 | Characterize farm types in costal ecosystem | FT = f(education index, cop-diversification index, cost of cultivation, gross return, net return, benefit ratio) | 5 |
| 6 | To cluster farmer types based on the preference for genetic improvement in cow traits | FT = f(socio-economic profile, farming practice, geographic location, attitude towards breeding and genetic evaluation tools, state preference of farmer) | 6 |
| 7 | To understand the diversity within farmers to plan efficiently for on-farm innovation | FT = f (biophysical resources, physical resources, socio-economic, system management) | 7 |
| 8 | To access the diversity in production strategies by smallholder rice farmers | FT = f (Total rice production, cultivated area, total work carried on farm, family size, proportion consumed, sold and stored per farm, yield) | 8 |
| 9 | To explore the link between agriculture adaptation strategies and | FT = f (food security and sufficiency, assets, land and labor force, ma | 9 |
| 10 | To evaluate the sustainability of different farm types | FT = f (social, economic and environmental) | 10 |
| 11 | To characterize the diversity in smallholder farmers to understand the type-specific constraints and opportunities for innovation | FT = f (household, labor, land use, livestock ownership and income) | 11 |
| 12 | To investigate the differences in willingness to diversity in future for identified farm types | FT = f (social and demographic household characteristics, income, characteristics of the agricultural holding, farm structure, and organization, specific variables regarding income diversification) | 12 |

**Table S.2: Description of the survey villages**

| **Chakrata tehsil (Dehradun)** | | | | | | | |
| --- | --- | --- | --- | --- | --- | --- | --- |
| **Village** | **Total household*** | **Sampled household**** | **Forest area (%)** | **Net Sown Area (%)** | **Irrigated area (%)** | **Agriculture population (%)** | **Crop diversity** |
| Manjhgaon | 59 | 18 | 0.00 | 28.24 | 7.72 | 41.09 | **Food crops**: Wheat, barley, maize, paddy  **Pulses:** Rajma, lentil  Oil seed: Mustard  **Cash crops:** Potato, tomato, cauliflower, onion, pea, ginger, capsicum, colocasia, lady finger  **Fruits:** Walnut, apricot, plum, peach, apple |
| Kurar | 81 | 24 | 8.59 | 23.40 | 6.18 | 69.07 |  |
| Jashta | 75 | 26 | 14.33 | 18.14 | 10.58 | 39.62 |  |
| Samog | 63 | 19 | 22.08 | 14.01 | 28.14 | 26.87 |  |
| Siribarkoti | 81 | 24 | 9.40 | 32.57 | 5.90 | 78.55 |  |
| **Bhikiyasain tehsil (Almora)** | | | | | | | |
| Babalia | 62 | 19 | 5.26 | 45.02 | 94.83 | 48.18 | **Food crops**: Paddy, wheat, barley, fingermillet (*mandua*), Chaulai  **Pulses**: Soyabean (*bhatt*), lentil, urad  **Oil seed**: Mustard, urad, gahat  **Cash crops**: Potatoes, chillies, turmeric, capsicum  **Fruits**: Mango, lime |
| Palpur | 83 | 25 | 18.04 | 76.39 | 64.66 | 45.12 |  |
| Kotesari | 120 | 36 | 16.39 | 50.12 | 97.93 | 49.31 |  |
| Walmara | 98 | 29 | 8.79 | 19.77 | 31.60 | 75.18 |  |
| Titari | 133 | 34 | 20.07 | 40.41 | 14.42 | 51.64 |  |

* Total household number did not match with census information

**30% sampling intensity

(Source: Census of India, 2011)

**Table S.3: Crop calendar of Chakrata and Bhikiyasain Tehsil, Uttarakhand**

| **Crops** | **Jan*** | **Feb** | **Mar** | **Apr** | **May** | **Jun** | **Jul*** | **Aug*** | **Sep*** | | **Oct** | | **Nov** | **Dec*** |
| --- | --- | --- | --- | --- | --- | --- | --- | --- | --- | --- | --- | --- | --- | --- |
|  | | | | | | | |  | | Sowing | |  | Harvesting | |
| **Cereals** | | | | | | | | | | | | | | |
| Paddy |  |  |  |  |  |  |  |  |  | |  | |  |  |
| Wheat |  |  |  |  |  |  |  |  |  | |  | |  |  |
| Millets |  |  |  |  |  |  |  |  |  | |  | |  |  |
| Ramdana |  |  |  |  |  |  |  |  |  | |  | |  |  |
| **Vegetables** | | | | | | | | | | | | | | |
| Potato |  |  |  |  |  |  |  |  |  | |  | |  |  |
| Tomato |  |  |  |  |  |  |  |  |  | |  | |  |  |
| Onion |  |  |  |  |  |  |  |  |  | |  | |  |  |
| Pea |  |  |  |  |  |  |  |  |  | |  | |  |  |
| Cauliflower |  |  |  |  |  |  |  |  |  | |  | |  |  |
| Cabbage |  |  |  |  |  |  |  |  |  | |  | |  |  |
| Lady finger |  |  |  |  |  |  |  |  |  | |  | |  |  |
| Colocasia(arbi) | |  |  |  |  |  |  |  |  | |  | |  |  |
| Capsicum |  |  |  |  |  |  |  |  |  | |  | |  |  |
| **Pulses** | | | | | | | | | | | | | | |
| Urad |  |  |  |  |  |  |  |  |  | |  | |  |  |
| Rajma |  |  |  |  |  |  |  |  |  | |  | |  |  |
| Gahat |  |  |  |  |  |  |  |  |  | |  | |  |  |
| Soyabeen |  |  |  |  |  |  |  |  |  | |  | |  |  |
| Masur |  |  |  |  |  |  |  |  |  | |  | |  |  |
| **Spices** | | | | | | | | | | | | | | |
| Coriander |  |  |  |  |  |  |  |  |  | |  | |  |  |
| Turmeric |  |  |  |  |  |  |  |  |  | |  | |  |  |
| Ginger |  |  |  |  |  |  |  |  |  | |  | |  |  |
| Chilly |  |  |  |  |  |  |  |  |  | |  | |  |  |
| Garlic |  |  |  |  |  |  |  |  |  | |  | |  |  |
| **Fruits** | | | | | | | | | | | | | | |
| Peach |  |  |  |  |  |  |  |  |  | |  | |  |  |
| Walnut |  |  |  |  |  |  |  |  |  | |  | |  |  |
| Pear |  |  |  |  |  |  |  |  |  | |  | |  |  |
| Apple |  |  |  |  |  |  |  |  |  | |  | |  |  |
| Palm(chulu) |  |  |  |  |  |  |  |  |  | |  | |  |  |
| Mango |  |  |  |  |  |  |  |  |  | |  | |  |  |

* July, August and September are the summer rainfall months and winter rainfall is received in the month of December and January

**Table S.4: Eigenvalues and percentage variance explained by 13 dimensions using FAMD output**

|  | **Eigen value** | **Variance explained (%)** | **Cumulative variance (%)** |
| --- | --- | --- | --- |
| Dim 1 | 6.63 | 16.26 | 16.18 |
| Dim 2 | 4.57 | 11.80 | 28.06 |
| Dim 3 | 2.38 | 5.81 | 33.13 |
| Dim 4 | 1.97 | 4.81 | 37.94 |
| Dim 5 | 1.80 | 4.40 | 42.33 |
| Dim 6 | 1.59 | 3.89 | 46.22 |
| Dim 7 | 1.45 | 3.53 | 49.75 |
| Dim 8 | 1.39 | 3.38 | 53.13 |
| Dim 9 | 1.28 | 3.13 | 56.26 |
| Dim 10 | 1.21 | 2.96 | 59.22 |
| Dim 11 | 1.11 | 2.71 | 61.92 |
| Dim 12 | 1.08 | 2.63 | 64.55 |
| Dim 13 | 1.02 | 2.50 | 67.05 |

**Table S.5: Variable loadings of different variables and categories on the resultant 13 factors**

|  | Dim.1 | Dim.2 | Dim.3 | Dim.4 | Dim.5 | Dim.6 | Dim.7 | Dim.8 | Dim.9 | Dim.10 | Dim.11 | Dim.12 | Dim.13 |
| --- | --- | --- | --- | --- | --- | --- | --- | --- | --- | --- | --- | --- | --- |
| Age | 0.026 | 0.078 | 0.035 | 0.037 | 0.036 | 0.012 | 0.026 | 0.005 | 0.206 | 0.166 | 0.006 | 0.103 | 0.003 |
| Tot_Land | 0.291 | 0.215 | 0.089 | 0.085 | 0.021 | 0.020 | 0.011 | 0.001 | 0.015 | 0.032 | 0.013 | 0.002 | 0.000 |
| Per_Irr | 0.035 | 0.095 | 0.157 | 0.042 | 0.283 | 0.004 | 0.045 | 0.000 | 0.008 | 0.001 | 0.034 | 0.000 | 0.022 |
| Per_Abn | 0.063 | 0.151 | 0.010 | 0.126 | 0.171 | 0.062 | 0.018 | 0.022 | 0.000 | 0.043 | 0.000 | 0.026 | 0.044 |
| TLU | 0.611 | 0.068 | 0.002 | 0.002 | 0.003 | 0.000 | 0.002 | 0.030 | 0.006 | 0.002 | 0.007 | 0.004 | 0.003 |
| Urea | 0.109 | 0.065 | 0.142 | 0.114 | 0.077 | 0.001 | 0.032 | 0.010 | 0.010 | 0.005 | 0.000 | 0.000 | 0.004 |
| Fam_Lab | 0.512 | 0.028 | 0.010 | 0.001 | 0.002 | 0.000 | 0.060 | 0.063 | 0.007 | 0.001 | 0.018 | 0.004 | 0.008 |
| HH_Size | 0.570 | 0.031 | 0.006 | 0.005 | 0.008 | 0.005 | 0.025 | 0.029 | 0.009 | 0.007 | 0.001 | 0.002 | 0.004 |
| Land_Ten | 0.168 | 0.205 | 0.502 | 0.042 | 0.106 | 0.064 | 0.084 | 0.041 | 0.132 | 0.029 | 0.007 | 0.046 | 0.011 |
| Gender | 0.094 | 0.008 | 0.011 | 0.053 | 0.025 | 0.003 | 0.145 | 0.271 | 0.036 | 0.003 | 0.015 | 0.002 | 0.033 |
| Edu | 0.049 | 0.278 | 0.165 | 0.255 | 0.124 | 0.275 | 0.190 | 0.507 | 0.256 | 0.149 | 0.258 | 0.426 | 0.435 |
| Sat_Far | 0.151 | 0.238 | 0.014 | 0.001 | 0.026 | 0.010 | 0.006 | 0.013 | 0.079 | 0.076 | 0.004 | 0.028 | 0.003 |
| Tar_Far | 0.015 | 0.487 | 0.003 | 0.098 | 0.000 | 0.069 | 0.008 | 0.012 | 0.000 | 0.002 | 0.012 | 0.000 | 0.001 |
| Plough | 0.501 | 0.433 | 0.057 | 0.160 | 0.013 | 0.028 | 0.011 | 0.005 | 0.022 | 0.010 | 0.056 | 0.004 | 0.035 |
| Cas | 0.560 | 0.464 | 0.054 | 0.066 | 0.050 | 0.456 | 0.081 | 0.041 | 0.105 | 0.058 | 0.102 | 0.017 | 0.047 |
| HH_Eco | 0.035 | 0.466 | 0.007 | 0.010 | 0.000 | 0.036 | 0.010 | 0.017 | 0.063 | 0.010 | 0.000 | 0.000 | 0.001 |
| Food_Suf | 0.244 | 0.458 | 0.269 | 0.216 | 0.278 | 0.240 | 0.345 | 0.063 | 0.004 | 0.140 | 0.177 | 0.090 | 0.073 |
| Hire_Lab | 0.045 | 0.279 | 0.107 | 0.005 | 0.023 | 0.027 | 0.008 | 0.017 | 0.052 | 0.038 | 0.006 | 0.084 | 0.000 |
| Wag_Lab | 0.163 | 0.209 | 0.001 | 0.019 | 0.076 | 0.057 | 0.005 | 0.012 | 0.015 | 0.031 | 0.012 | 0.009 | 0.001 |
| Soc_Bond | 0.363 | 0.082 | 0.148 | 0.237 | 0.114 | 0.038 | 0.208 | 0.031 | 0.108 | 0.308 | 0.255 | 0.136 | 0.162 |
| Crop | 0.778 | 0.373 | 0.483 | 0.287 | 0.290 | 0.064 | 0.107 | 0.115 | 0.095 | 0.026 | 0.094 | 0.101 | 0.123 |
| Credit | 0.400 | 0.009 | 0.019 | 0.017 | 0.015 | 0.021 | 0.002 | 0.009 | 0.030 | 0.003 | 0.020 | 0.002 | 0.000 |
| Market | 0.722 | 0.003 | 0.011 | 0.056 | 0.026 | 0.000 | 0.005 | 0.000 | 0.000 | 0.007 | 0.007 | 0.001 | 0.003 |

**Table S.6: Cluster specific mean and standard deviation of continuous variables that significantly characterize the portioning of the five different farmer types**

| Continuous variables | Type 1 | Type 2 | Type 3 | Type 4 | Type 5 |
| --- | --- | --- | --- | --- | --- |
| TLU | 1.82±1.39 | 12.25±6.00 | 9.01±5.17 | 2.80±2.18 | 2.11±1.72 |
| Tot_Land | 0.67±0.46 | 1.13±0.65 | 0.17±0.15 | 0.24±0.27 | 0.01± 0.01 |
| HH_Size | 5.06±1.48 | 13.81±6.37 | 9.56±4.32 | 4.97±1.37 | 4.87±1.46 |
| Fam_Lab | 2.51±0.68 | 5.27±2.31 | 4.39±2.10 | 2.14±0.81 | 1.13±1.10 |
| Urea | 12.60±11.49* | 18.41±12.64* | 52.87±30.12 | 12.97±8.07 | 0.00 |
| Per_Irr | 56.91±25.48 | 33.43±19.54 | 13.01±18.73 | 21.92±5.69* | 6.25± 0.18 |
| Per_Abn | 19.69±21.13 | 4.62±9.20 | 3.75±5.02 | 18.01±10.47 | 0.00 |
| Age | 58.24±13.79 | 45.75±14.02* | 41.39±9.95 | 50.05±16.73* | 37.02±20.19* |

* Non-significant p-value (p-value>0.05)

**Table S.7: Specificities (Cla/Mod) and homogeneities (Mod/Cla) for five farmer types listing the statistically significant categories of categorical variables that characterize the farmer type cluster. P-values for all the categories was less than 0.05**

| **Variable (category)** | **Cla/Mod** | **Mod/Cla** | **Global** | **v.test** |
| --- | --- | --- | --- | --- |
| Type 1 | | | | |
| Tar_Far=Tra_Yes | 63.64 | 96.55 | 18.26 | 10.14 |
| Plough=Plo_PT | 100.00 | 72.41 | 8.71 | 10.11 |
| Food_Suf=Six | 60.53 | 79.31 | 15.77 | 8.36 |
| HH_Eco=APL | 30.85 | 100.00 | 39.00 | 7.50 |
| Cas=General | 29.90 | 100.00 | 40.25 | 7.36 |
| Crop=MFLC | 48.48 | 55.17 | 13.69 | 5.76 |
| Hire_Lab=Hir_Yes | 56.52 | 44.83 | 9.54 | 5.50 |
| Sat_Far=Lik_Yes | 23.42 | 89.66 | 46.06 | 5.14 |
| Wag_Lab=Lab_no | 18.35 | 100.00 | 65.56 | 4.77 |
| Land_Ten=Self_Land | 14.36 | 100.00 | 83.82 | 2.86 |
| Edu=Intermediate | 37.50 | 20.69 | 6.64 | 2.69 |
| Edu=Bachelor | 50.00 | 13.79 | 3.32 | 2.60 |
| Soc_Bond=Soc_M | 24.39 | 34.48 | 17.01 | 2.43 |
| **Type 2** | | | | |
| Edu=Masters | 66.67 | 6.90 | 1.24 | 2.05 |
| Market=Mar_Yes | 38.05 | 100.00 | 46.89 | 8.42 |
| Credit=Cre_Yes | 50.00 | 86.05 | 30.71 | 8.36 |
| Plough=Plo_Ox | 31.16 | 100.00 | 57.26 | 7.09 |
| Sat_Far=Lik_Yes | 35.14 | 90.70 | 46.06 | 6.69 |
| Soc_Bond=Soc_VH | 37.35 | 72.09 | 34.44 | 5.54 |
| Wag_Lab=Lab_no | 26.58 | 97.67 | 65.56 | 5.48 |
| Food_Suf=Nine | 100.00 | 16.28 | 2.90 | 4.62 |
| Land_Ten=Self_Land | 21.29 | 100.00 | 83.82 | 3.70 |
| Tar_Far=Tra_No | 21.32 | 97.67 | 81.74 | 3.31 |
| Food_Suf=Six | 36.84 | 32.56 | 15.77 | 3.05 |
| Hire_Lab=Hir_Yes | 43.48 | 23.26 | 9.54 | 2.99 |
| Edu=Illiterate | 26.17 | 65.12 | 44.40 | 2.97 |
| Gender=M | 20.69 | 97.67 | 84.23 | 2.93 |
| **Type 3** | | | | |
| Market=Mar_Yes | 44.25 | 94.34 | 46.89 | 8.25 |
| Plough=Plo_Ox | 37.68 | 98.11 | 57.26 | 7.55 |
| Crop=Cash_Cr | 91.30 | 39.62 | 9.54 | 7.48 |
| HH_Eco=BPL | 34.69 | 96.23 | 61.00 | 6.54 |
| Soc_Bond=Soc_VH | 38.55 | 60.38 | 34.44 | 4.36 |
| Crop=MCLF | 40.58 | 52.83 | 28.63 | 4.21 |
| Tar_Far=Tra_No | 26.40 | 98.11 | 81.74 | 3.92 |
| Cas=SC | 39.13 | 50.94 | 28.63 | 3.89 |
| Cas=ST | 37.68 | 49.06 | 28.63 | 3.57 |
| Sat_Far=Lik_No | 30.00 | 73.58 | 53.94 | 3.26 |
| Food_Suf=Nil | 35.21 | 47.17 | 29.46 | 3.08 |
| Hire_Lab=Hir_No | 24.31 | 100.00 | 90.46 | 3.04 |
| **Type 4** | | | | |
| Market=Mar_No | 78.13 | 100.00 | 53.11 | 13.64 |
| Crop=Food_Cr | 82.52 | 85.00 | 42.74 | 11.59 |
| Cas=General | 70.10 | 68.00 | 40.25 | 7.43 |
| Plough=Plo_No | 71.95 | 59.00 | 34.02 | 6.88 |
| Credit=Cre_No | 54.49 | 91.00 | 69.29 | 6.41 |
| Hire_Lab=Hir_No | 45.87 | 100.00 | 90.46 | 4.76 |
| Gender=F | 100.00 | 29.00 | 15.77 | 4.69 |
| Land_Ten=Self_Rent_Land | 96.96 | 20.00 | 9.54 | 4.63 |
| Soc_Bond=Soc_H | 67.86 | 38.00 | 23.24 | 4.51 |
| Wag_Lab=Lab_yes | 59.04 | 49.00 | 34.44 | 3.96 |
| Sat_Far=Lik_No | 52.31 | 68.00 | 53.94 | 3.68 |
| HH_Eco= BPL | 53.19 | 50.00 | 39.00 | 2.92 |
| **Type 5** | | | | |
| Land_Ten=Rent_Land | 93.75 | 93.75 | 6.64 | 9.58 |
| Crop=No_Cr | 92.31 | 75.00 | 5.39 | 8.14 |
| Plough=Plo_No | 19.51 | 100.00 | 34.02 | 5.71 |
| Cas=SC | 21.74 | 93.75 | 28.63 | 5.56 |
| Soc_Bond=Soc_VL | 27.66 | 81.25 | 19.50 | 5.46 |
| Wag_Lab=Lab_yes | 16.87 | 87.50 | 34.44 | 4.42 |
| Market=Mar_No | 12.50 | 100.00 | 53.11 | 4.21 |
| Sat_Far=Lik_No | 12.31 | 100.00 | 53.94 | 4.15 |
| Food_Suf=Nil | 16.90 | 75.00 | 29.46 | 3.80 |
| HH_Eco=BPL | 10.88 | 100.00 | 61.00 | 3.65 |
| Credit=Cre_No | 9.58 | 100.00 | 69.29 | 3.06 |
| Tar_Far=Tra_No | 8.12 | 100.00 | 81.74 | 2.10 |

***References***

1. Tittonell, P., Leffelaar, P. A., Vanlauwe, B., van Wijk, M. T. & Giller, K. E. Exploring diversity of crop and soil management within smallholder African farms: A dynamic model for simulation of N balances and use efficiencies at field scale. *Agric. Syst.* **91**, 71–101 (2006).
2. Iraizoz, B., Gorton, M. & Davidova, S. Segmenting farms for analysing agricultural trajectories: A case study of the Navarra region in Spain. *Agric. Syst.* **93**, 143–169 (2007).
3. Bidogeza, J. C., Berentsen, P. B. M., Graaff, J. & Oude Lansink, a. G. J. M. A typology of farm households for the Umutara Province in Rwanda. *Food Secur.* **1**, 321–335 (2009).
4. Sakané, N., Becker, M., Langensiepen, M. & Van Wijk, M. T. Typology of smallholder production systems in small east-African wetlands. *Wetlands* **33**, 101–116 (2013).
5. Goswami, R., Chatterjee, S. & Prasad, B. Farm types and their economic characterization in complex agro-ecosystems for informed extension intervention: study from coastal West Bengal, India. *Agric. Food Econ.* **2**, 5 (2014).
6. Martin-Collado, D., Soini, K., Mäki-Tanila, A., Toro, M. A. & Díaz, C. Defining farmer typology to analyze the current state and development prospects of livestock breeds: The Avile??a-Negra Ib??rica beef cattle breed as a case study. *Livest. Sci.* **169**, 137–145 (2014).
7. Cortez-Arriola, J. *et al.* Leverages for on-farm innovation from farm typologies? An illustration for family-based dairy farms in north-west Michoac??n, Mexico. *Agric. Syst.* **135**, 66–76 (2015).
8. Chenoune, R., Belhouchette, H., Paloma, S. G. y & Capillon, A. Assessing the diversity of smallholder rice farms production strategies in Sierra Leone. *NJAS - Wageningen J. Life Sci.* **76**, 7–19 (2016).
9. Douxchamps, S. *et al.* Linking agricultural adaptation strategies, food security and vulnerability: evidence from West Africa. *Reg. Environ. Chang.* **16**, 1305–1317 (2016).
10. Haileslassie, A. *et al.* Empirical evaluation of sustainability of divergent farms in the dryland farming systems of India. *Ecol. Indic.* **60**, 710–723 (2016).
11. Kuivanen, K. S. *et al.* Characterising the diversity of smallholder farming systems and their constraints and opportunities for innovation: A case study from the Northern Region, Ghana. *NJAS - Wageningen J. Life Sci.* **78**, 153–166 (2016).
12. Weltin, M. *et al.* Analysing behavioural differences of farm households: An example of income diversification strategies based on European farm survey data. *Land use policy* **62**, 172–184 (2017).
